# Socioeconomic inequality in the prevalence of low birth weight and its associated determinants in Bangladesh

**DOI:** 10.1371/journal.pone.0276718

**Published:** 2022-10-27

**Authors:** Md. Jahangir Alam, Md. Merajul Islam, Md. Maniruzzaman, N. A. M. Faisal Ahmed, Most. Tawabunnahar, Md. Jahanur Rahman, Dulal Chandra Roy, Janardhan Mydam

**Affiliations:** 1 Department of Statistics, University of Rajshahi, Rajshahi, Bangladesh; 2 Department of Statistics, Jatiya Kabi Kazi Nazrul Islam University, Mymensingh, Bangladesh; 3 Statistics Discipline, Khulna University, Khulna, Bangladesh; 4 Institute of Education and Research, University of Rajshahi, Rajshahi, Bangladesh; 5 Division of Neonatology, Department of Pediatrics, John H. Stroger, Jr. Hospital of Cook County, Chicago, IL, United States of America; 6 Department of Pediatrics, Rush Medical Center, Chicago, IL, United States of America; 7 Division of Neonatology, Department of Pediatrics, College of Medicine, University of Illinois at Chicago, Chicago, IL, United States of America; Universidade Federal do Rio de Janeiro, BRAZIL

## Abstract

**Background and objective:**

Low birth weight (LBW) is a major risk factor of child mortality and morbidity during infancy (0–3 years) and early childhood (3–8 years) in low and lower-middle-income countries, including Bangladesh. LBW is a vital public health concern in Bangladesh. The objective of the research was to investigate the socioeconomic inequality in the prevalence of LBW among singleton births and identify the significantly associated determinants of singleton LBW in Bangladesh.

**Materials and methods:**

The data utilized in this research was derived from the latest nationally representative Bangladesh Demographic and Health Survey, 2017–18, and included a total of 2327 respondents. The concentration index (C-index) and concentration curve were used to investigate the socioeconomic inequality in LBW among the singleton newborn babies. Additionally, an adjusted binary logistic regression model was utilized for calculating adjusted odds ratio and p-value (<0.05) to identify the significant determinants of LBW.

**Results:**

The overall prevalence of LBW among singleton births in Bangladesh was 14.27%. We observed that LBW rates were inequitably distributed across the socioeconomic groups (C-index: -0.096, 95% confidence interval: [-0.175, -0.016], *P* = 0.029), with a higher concentration of LBW infants among mothers living in the lowest wealth quintile (poorest). Regression analysis revealed that maternal age, region, maternal education level, wealth index, height, age at 1st birth, and the child’s aliveness (alive or died) at the time of the survey were significantly associated determinants of LBW in Bangladesh.

**Conclusion:**

In this study, socioeconomic disparity in the prevalence of singleton LBW was evident in Bangladesh. Incidence of LBW might be reduced by improving the socioeconomic status of poor families, paying special attention to mothers who have no education and live in low-income households in the eastern divisions (e.g., Sylhet, Chittagong). Governments, agencies, and non-governmental organizations should address the multifaceted issues and implement preventive programs and policies in Bangladesh to reduce LBW.

## Introduction

Low birth weight (LBW) is a leading public health concern. It is a vital risk factor of perinatal survival, infant and child mortality, and morbidity in infancy and early childhood (3–8 years), and it contributes prominently to the overall burden of infant mortality [1, 2]. Infants with LBW may have digestive and breathing problems and complications in eating, gaining weight, and fighting off infections compared with normal birth weight infants [3]. As LBW infants grow into adulthood, they may have mental retardation, developmental disorders, physical disabilities, exhaustion/fatigue, depression, and other psychiatric conditions. They also have an increased risk of non-communicable diseases including hypertension, diabetes, chronic snoring (sleeping-disordered breathing), and cardiovascular disease [4–9]. Every year 15% to 20% of all live births have LBW across the globe [10, 11], of which 91% are from lower-middle-income countries (LMICs) [12] and around 50% occur in Bangladesh and India [12, 13]. Approximately 80% of annual newborn deaths are linked to LBW delivery globally [12, 14–17]. In Bangladesh, 38% of all newborn deaths are related to LBW [18]. The prevalence of LBW in Bangladesh was 17.7% in 2011 [19], 20% in 2014 [20], and 16% in 2017 [21]. Although the prevalence of LBW decreased in Bangladesh, it is still higher compared to most of the developed and developing countries [13]. To reduce the prevalence of LBW in LMICs, it is important to identify the most significant contributing factors. Socioeconomic factors such as wealth index, education, family income, occupation, and family size are prominent determinants of LBW [22, 23]. Pregnant women who live in the poor households (i.e., households with low socioeconomic status) may have less access to health care services, and greater food and nutritional insecurity compared to women living in wealthy households (i.e., households with high socioeconomic status), placing them at higher risk for LBW infants [24, 25]. Therefore, it is important to further investigate the role of socioeconomic inequality and its associated determinants in Bangladesh.

To the best of our knowledge, no existing study investigated the socioeconomic inequality in the prevalence of LBW using the nationally representative BDHS data. Thus, the objectives of the present study were to statistically investigate the socioeconomic inequality in the prevalence of LBW in Bangladesh and identify the significantly associated determinants of LBW, using data from the most recent BDHS (2017–2018).

## Materials and methods

### Data source

**Fig 1:**
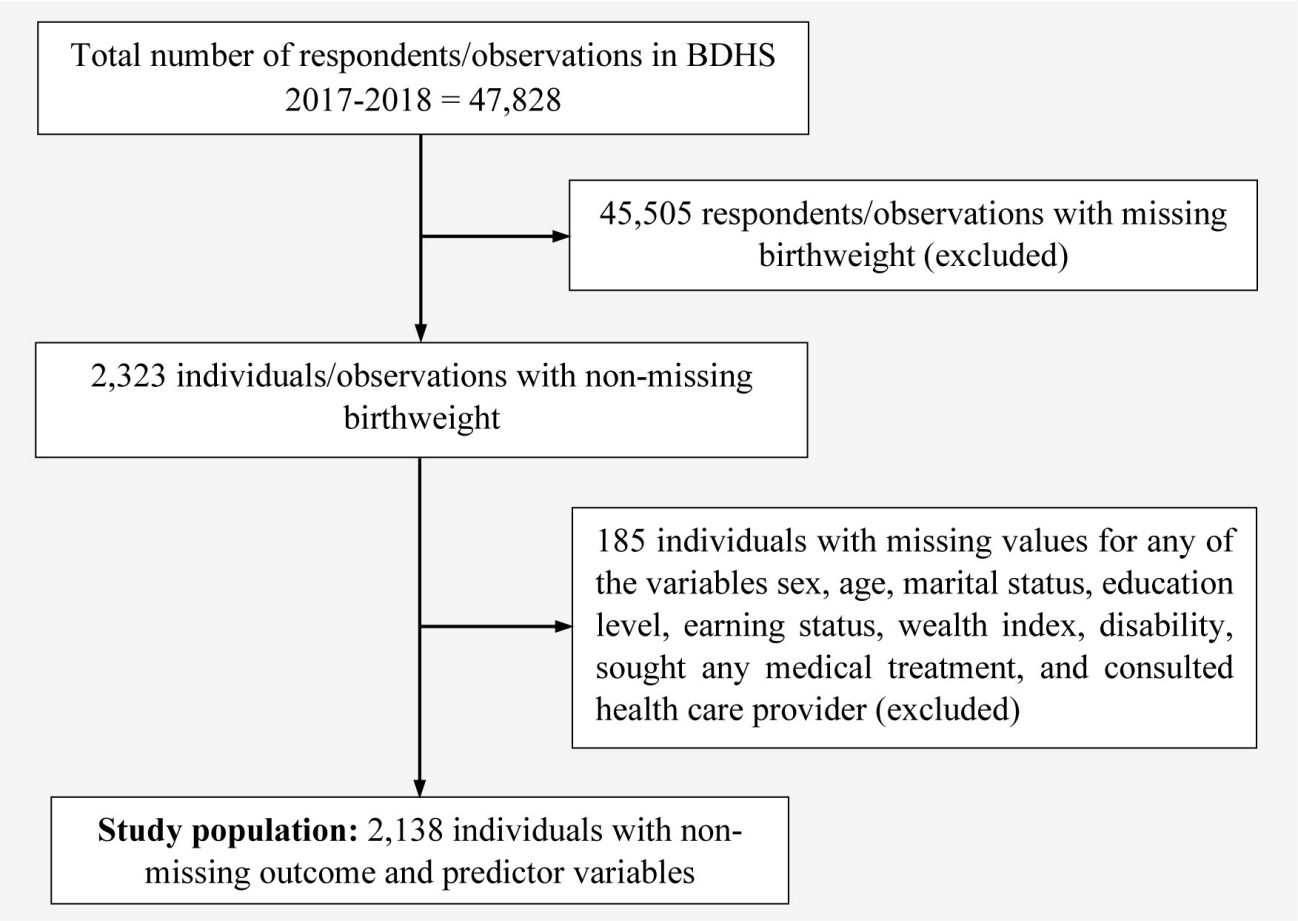
Study flowchart of inclusion/exclusion of observations.

The dataset utilized in this study was taken from Bangladesh Demographic and Health Survey (BDHS), 2017–18 [21]. The BDHS data was collected through two-stage stratified cluster sampling. In the 1^st^ stage, 675 enumeration areas (EAs) were selected via probability proportional sampling, wherein 250 were urban and 425 rural areas. In the 2^nd^ stage, sorting the taken households and provided a complete sampling frame. A systematic random sample of 30 households was chosen from each EA in the 2^nd^ stage to estimate the key demographic and health-related indicators. A total of 20,250 residential households were chosen to participate in face-to-face interviews with questionnaires. About 20,100 ever-married women with an age range of 15–49 were expected to complete the interviews [21]. Each respondent was asked to give the overall birth history for births during the survey period, and the birth weight was measured in grams. A total of 47,828 respondents provided their birth information in BDHS 2017–18. Implementing the sample weight variable, excluding unusual observations and missing values, 2,138 observations were selected for the final analysis. A brief description of the data extraction procedure is depicted in Fig 1.

### Ethical approval

The present study utilized current public domain survey datasets, which are freely accessible online; that is why it does not require any additional ethical approval. A detailed description of the ethical procedures followed by the DHS program (https://dhsprogram.com) can be found in the BDHS reports [21].

### Dependent/Outcome variable

The outcome variable in this study was LBW, measured by grams based on the WHO cutoff (birth weight < 2500 g), and recorded a binary response variable with a membership class label: LBW and non-LBW [26]. The membership class label was coded as “1” for LBW and “0” for non-LBW.

### Explanatory/Independent variables

The explanatory variables included in this study are based on the earlier research papers on literature [5, 27–34]. The explanatory variables included demographic characteristics: maternal age (≤20, 21–30, 31–40, ≥40), residence (urban, rural), region (Barisal, Chittagong, Dhaka, Khulna, Mymensingh, Rajshahi, Rangpur, Sylhet), religion (Muslim, non-Muslim), and sex of child (male, female); socioeconomic characteristics: mother’s education level (no education, primary, secondary, higher), husband/partner’s education level (no education, primary, secondary, higher), and wealth index (poorest, poorer, middle, richer, richest); physical and medical information: height, weight, maternal nutritional status (underweight, normal, overweight, obese), parity (≤3, >3), age at first birth (<15, 15–25, >25), marriage to 1^st^ birth interval(≤30, >30), antenatal care (ANC) initiation at 1^st^ trimester (yes, no), number of antenatal visits (<4, ≥4), during pregnancy iron tablet (yes, no), place of delivery (home, government sector, private sector, NGO and others), delivery by CS (yes, no), and child is alive (yes, no); environmental characteristic: toilet facility (hygienic, unhygienic); and exposure to mass media: newspaper (yes, no), and television (yes, no). Based on WHO criteria, nutritional status of mothers was measured by body mass index (BMI) with underweight (BMI<18.5 kg/m^2^), normal (18.5≤BMI≤24.9 kg/m^2^), overweight (25≤BMI≤29.9 kg/m^2^), and obese (≥ 30.0 kg/m^2^).

### Statistical analysis

Data was prepared using the survey weights before the statistical analysis. In bivariate analysis, Pearson’s chi-squared test of independence [35–40] was implemented to examine the association between two categorical variables, and independent samples t-test was employed for determining the significant difference between the group means of the normally distributed data. Primarily, an unadjusted logistic regression (LR) model was performed to establish the strength of the associations between LBW and the explanatory variables and to calculate unadjusted/crude odds ratio (COR) along with 95% confidence interval (CI). Adjusted LR model with a stepwise forward selection method was employed to identify significantly associated risk factors for LBW [41, 42] and to calculate adjusted odds ratio (AOR) and it 95% CI. The explanatory variables with a p-value <0.05 from the bivariate analysis were included as independent variables in the LR models. The LR model was expressed by the following expression:

$$\ln\;\left(\frac{P(X)}{1‐P(X)}\right)=\beta_{0}+\sum_{i=1}^{14}\beta_{k}X_{k}+\varepsilon$$ (1)

where, X = (X_1_: maternal age, X_2_: division, X_3_: mother’s education level, X_4_: wealth index, X_5_: height, X_6_: weight, X_7_: parity, X_8_: age at 1^st^ birth, X_9_: antenatal care initiation at 1^st^ trimester, X_10_: number of antenatal visits, X_11_: delivery by CS, X_12_: child is alive, X_13_: toilets; X_14_: newspaper) represent explanatory variables; β_0_ is intercept; β = (β_1_,β_2_,…,β_14_) represents the regression coefficients; and ε denotes random error term. Odds ratio (OR) with 95% confidence intervals (CIs) was calculated aimed at assessing the directions as well as the strength of the effect of the explanatory variables. The explanatory variable with a p-value <0.05 was considered statistically significant for the determinants of LBW. Data processing/preparation and all statistical analyses were carried out by SAS 9.4 software (SAS Institute, Inc., Cary, North Carolina).

#### Concentration index

The concentration index (C-index) was computed to quantify the degree of socioeconomic inequality of newly born babies with LBW among singleton births. C-index is a well-known and suitable measurement for measuring socioeconomic inequality in health-related variables [43]. The CI was calculated using the following formula:

$$C‐\mathrm{index}=\frac{2}{\mu }\mathrm{cov}(y_{i},R_{i})$$ (2)

where, y_i_: LBW, μ: mean of LBW, R_i_: i^th^ individual’s fractional rank in the socioeconomic distribution [44]. The range of C-index lies between − 1 to + 1. If the curve displays above the line of equality, the value of C-index is negative and indicates a disproportionally concentration of inequality among the poor [45, 46]. If the curve falls below the line of equality, the value of C-index is positive, demonstrating a disproportionally concentrated inequality among the rich. There is no socioeconomic inequality when the value of C-index is zero. The larger absolute value of C-index explores the higher inequities among socioeconomic households. Concentration curve (CC) with p-value was also portrayed for a clear illustration.

## Results

### Baseline characteristics of the participants

Table 1 shows the descriptive statistics of the study variables. In this study, the study population consisted of 2,138 respondents/mothers (age:15–49 years) with 22 LBW-related explanatory variables. The average age of the mothers was 24.79±5.505 years, with an average height of 151.47±5.73, average weight of 53.61±10.90, and average BMI of 23.32±4.32. In the study population, the majority of the mothers were younger (age≤30 years), among which the proportion of 21–30 years old mothers was 53.18% followed by the mothers of age ≤20 years (35.36%). The highest proportion of mothers had normal BMI (55.75%), while the overweighted mothers had the 2^nd^ highest proportion (24.65%). More than half of the mothers were secondary educated (51.08%), whereas 2.67% of mothers had no education, 15.34% were primary literate, and 30.92% had higher degrees. About 52% of mothers initiated antenatal care during pregnancy at the 1^st^ trimester, and most of mothers delivered their babies by cesarean section (62.72%). In addition, a larger proportion of mothers had ≥4 ANC visits (68.01%), 15–25 years age at first birth (89.52%), hygienic toilet facility (82.79%) and lived in the Dhaka division (17.59%).

**Table 1:** Distribution of the risk factors by LBW using BDHS, 2017–18.

	Overall, n (%)	LBW, n (%)	Non-LBW, n (%)	P-value
**Total**	2138 (100%)	305 (14.27%)	1,833 (85.73%)	
**Maternal age (Years)**				
< = 20	756 (35.36)	118 (15.61)	638 (84.39)	0.023
21–30	1137 (53.18)	151 (13.28)	986 (86.72)	
31–40	233 (10.90)	31 (13.30)	202 (86.70)	
>40	12 (0.56)	5 (41.67)	7 (58.33)	
**Residence**				
Urban	931 (43.55)	126 (13.53)	805 (86.47)	0.395
Rural	1207 (56.45)	179 (14.83)	1028 (85.17)	
**Division**				
Barisal	188 (8.79)	25 (13.30)	163 (86.70)	0.014
Chittagong	312 (14.59)	60 (19.23)	252 (80.77)	
Dhaka	376 (17.59)	51 (13.56)	325 (86.44)	
Khulna	300 (14.03)	40 (13.33)	260 (86.67)	
Mymensingh	243 (11.37)	23 (9.47)	220 (90.53)	
Rajshahi	240 (11.23)	37 (15.42)	203 (84.58)	
Rangpur	292 (13.66)	33 (11.30)	259 (88.70)	
Sylhet	187 (8.75)	36 (19.25)	151 (80.75)	
**Religion**				
Muslim	1917 (89.66)	271 (14.14)	1646 (85.86)	0.615
Non-Muslim	221 (10.34)	34 (15.38)	187 (84.62)	
**Sex of child**				
Male	1159 (54.21)	152 (13.11)	1007 (86.89)	0.098
Female	979 (45.79)	153 (15.63)	826 (84.37)	
**Maternal education**				
No education	57 (2.67)	14 (24.56)	43 (75.44)	0.002
Primary	328 (15.34)	57 (17.38)	271 (82.62)	
Secondary	1092 (51.08)	164 (15.02)	928 (84.98)	
Higher	661 (30.92)	70 (10.59)	591 (89.41)	
**Husband education**				
No education	158 (7.39)	25 (15.82)	133 (84.18)	0.001
Primary	513 (23.99)	87 (16.96)	426 (83.04)	
Secondary	766 (35.83)	123 (16.06)	643 (83.94)	
Higher	701 (32.79)	70 (9.99)	631 (90.01)	
**Height, cm [mean (SD)]**	151.47 (5.73)	149.68 (5.66)	151.77 (5.69)	<0.001
**Weight, kg [mean (SD)]**	53.61 (10.90)	51.49 (11.04)	53.97 (10.84)	<0.001
**BMI**	23.32 (4.32)	22.90 (4.33)	23.40 (4.31)	0.063
Underweight	265 (12.39)	42 (15.85)	223 (84.15)	0.455
Normal	1192 (55.75)	174 (14.60)	1018 (85.40)	
Overweight	527 (24.65)	73 (13.85)	454 (86.15)	
Obese	154 (7.20)	16 (10.39)	138 (89.61)	
**Parity**				
≤3	2013 (94.15)	278 (13.81)	1735 (86.19)	0.016
>3	125 (5.85)	27 (21.60)	98 (78.40)	
**Age at 1**^**st**^ **birth (years)**				
<15	81 (3.79)	18 (22.22)	63 (77.78)	0.005
15–25	1914 (89.52)	277 (14.47)	1637 (85.53)	
>25	143 (6.69)	10 (6.99)	133 (93.01)	
**Marriage to 1**^**st**^ **birth interval**				
< = 30	1513 (70.77)	226 (14.94)	1287 (85.06)	0.167
>30	625 (29.23)	79 (12.64)	546 (87.36)	
**ANC initiation at 1**^**st**^ **trimester**				
Yes	1110 (51.92)	137 (12.34)	973 (87.66)	0.008
No	1028 (48.08)	168 (16.34)	860 (83.66)	
**Number of antenatal visits**				
<4	684 (31.99)	114 (16.67)	570 (83.33)	0.030
> = 4	1454 (68.01)	191 (13.14)	1263 (86.86)	
**During pregnancy iron tablet**				
Yes	1850 (86.53)	254 (13.73)	1596 (86.27)	0.073
No	288 (13.47)	51 (17.71)	237 (82.29)	
**Place of delivery**				
Home	198 (9.26)	32 (16.16)	166 (83.84)	0.178
Public sector	528 (24.70)	87 (16.48)	441 (83.52)	
Private sector	1235 (57.76)	162 (13.12)	1073 (86.88)	
NGO sector	175 (8.19)	23 (13.14)	152 (86.86)	
Other	2 (0.09)	1 (50.00)	1 (50.00)	
**Delivery by CS**				
Yes	1341 (62.72)	174 (12.98)	1167 (87.02)	0.027
No	797 (37.28)	131 (16.44)	666 (83.56)	
**Child is alive**				
Yes	2095 (97.99)	291 (13.89)	1804 (86.11)	<0.001
No	43 (2.01)	14 (32.56)	29 (67.44)	
**Toilet facility** ^**a**^				
Hygienic	1770 (82.79)	240 (13.56)	1530 (86.44)	0.041
Unhygienic	368 (17.21)	65 (17.66)	303 (82.34)	
**Newspaper**				
Yes	383 (17.91)	39 (10.18)	344 (89.82)	0.012
No	1755 (82.09)	266 (15.16)	1489 (84.84)	
**Television**				
Yes	1621 (75.82)	228 (14.07)	1393 (85.93)	0.639
No	517 (24.18)	77 (14.89)	440 (85.11)	

ANC: antenatal care, BMI: body mass index, CS: caesarean section, LBW: low birth weight, NGO: non-governmental organization, SD: standard deviation.

^a^ Hygienic toilet facility includes flush toilet, flush to piped sewer system, flush to septic tank, flush to pit latrine, flush to somewhere else, flush to unknow place, pit toilet latrine, ventilated improved pit latrine (VIP), pit latrine with slab and composting toilet. Unhygienic toilet facility includes all other toilet facilities that are not included under hygienic toilet facility (pit latrine without slab/open pit, no facility, no facility/bush/field, bucket toilet, hanging toilet/latrine and other).

**Fig 2:**
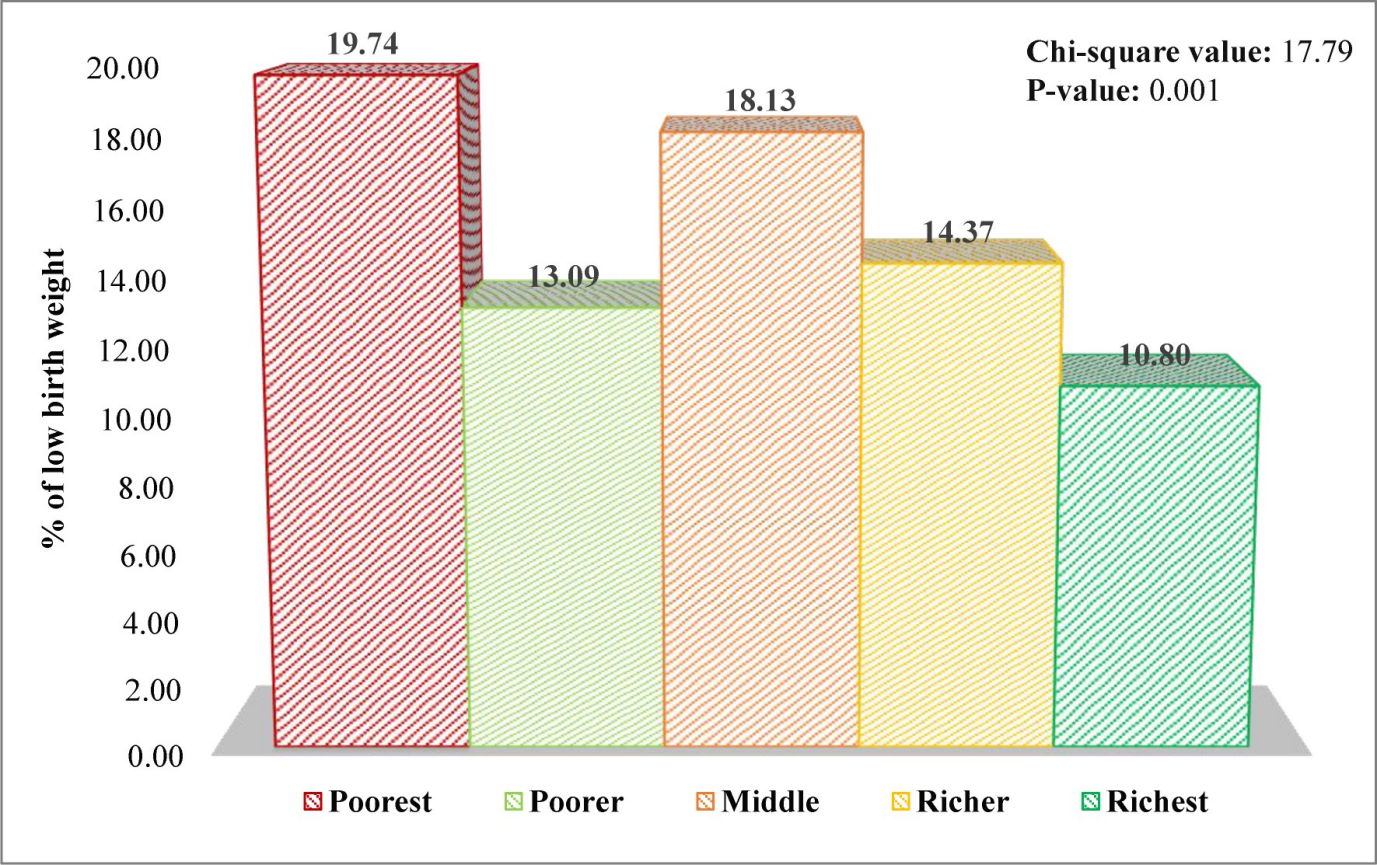
Prevalence of LBW among different groups of socioeconomic status.

In our study population, the overall prevalence of LBW was 14.27% (Table 1). The prevalence of LBW among different groups of socioeconomic status has been displayed in Fig 2. It shows that the prevalence of LBW was the highest among the poorest mothers (19.74%) compared to those who had middle (18.13%) and richest (10.80%) socioeconomic status. The p-value of the chi-squared test was 0.001, which indicates a highly significant association between socioeconomic status and LBW status.

The distribution of the explanatory variables by LBW status is presented in Table 1. The prevalence of LBW was higher among younger (age ≤20 years) and older (age > 40 years) mothers. The association between maternal age and LBW status was statistically significant (*P* = 0.023). The Sylhet division was found to have the highest prevalence rate of LBW (19.25%), while the Mymensingh division had the lowest LBW rate (9.47%). The division was significantly associated with LBW status. Non-educated mothers were found to have higher LBW babies (24.56%) compared to primary (17.38%), secondary (15.02%), and higher (10.59%) educated mothers. Parents’ education level and LBW were also statistically significant. Underweighted mothers exhibited the highest LBW rate (15.85%) followed by the mothers with normal BMI (14.60%). Mothers who gave birth to their first babies at age <15 years were found to have a higher prevalence of LBW (22.22%) than others (15–25 years: 14.47% and >25 years: 6.99%), exhibiting the statistically significant association between age at first birth and LBW status. Mothers who did not initiate ANC at the first trimester during their pregnancy period were found to have a significantly higher prevalence rate of LBW compared to their counterparts (16.34% versus 12.34%, *P* = 0.008). The prevalence of LBW was significantly higher among the mother who had < 4 ANC visits than those who had >4 ANC visits (16.67% versus 13.14%, *P* = 0.030). This indicates that number of ANC visits was significantly associated with LBW. Mothers who had normal delivery (i.e., spontaneous vaginal delivery) exhibited higher prevalence rate of LBW in a comparison with those who delivered by cesarean (16.44% versus 12.98%) and the association of LBW with model of delivery was statistically significant (*P* = 0.027). Families having no toilets facilities had a significantly higher prevalence of LBW than the families with hygienic toilets facilities (17.66% versus 13.56%, *P* = 0.041), which indicates a significant association between toile facility and LBW. In terms of exposure to mass media, not reading newspaper was significantly associated with LBW (read newspaper: 10.18% versus not read newspaper: 15.16%, *P* = 0.012). Area of residence, religion, sex of the child, mother’s BMI, marriage to 1^st^ birth interval, wanted pregnancy, taking iron tablet during pregnancy, place of delivery, and watching television were not significantly associated with LBW.

**S1 Table** demonstrated that the predictors maternal age, residence, division, parental education, BMI, parity, age at first birth, Marriage to 1^st^ birth interval, ANC initiation at 1^st^ trimester, number of antenatal visits, taking iron tablet during pregnancy, place of delivery, mode of delivery, toilet facility, reading newspaper, watching television were significantly associated with the socio-economic status (*P*<0.005).

**Fig 3:**
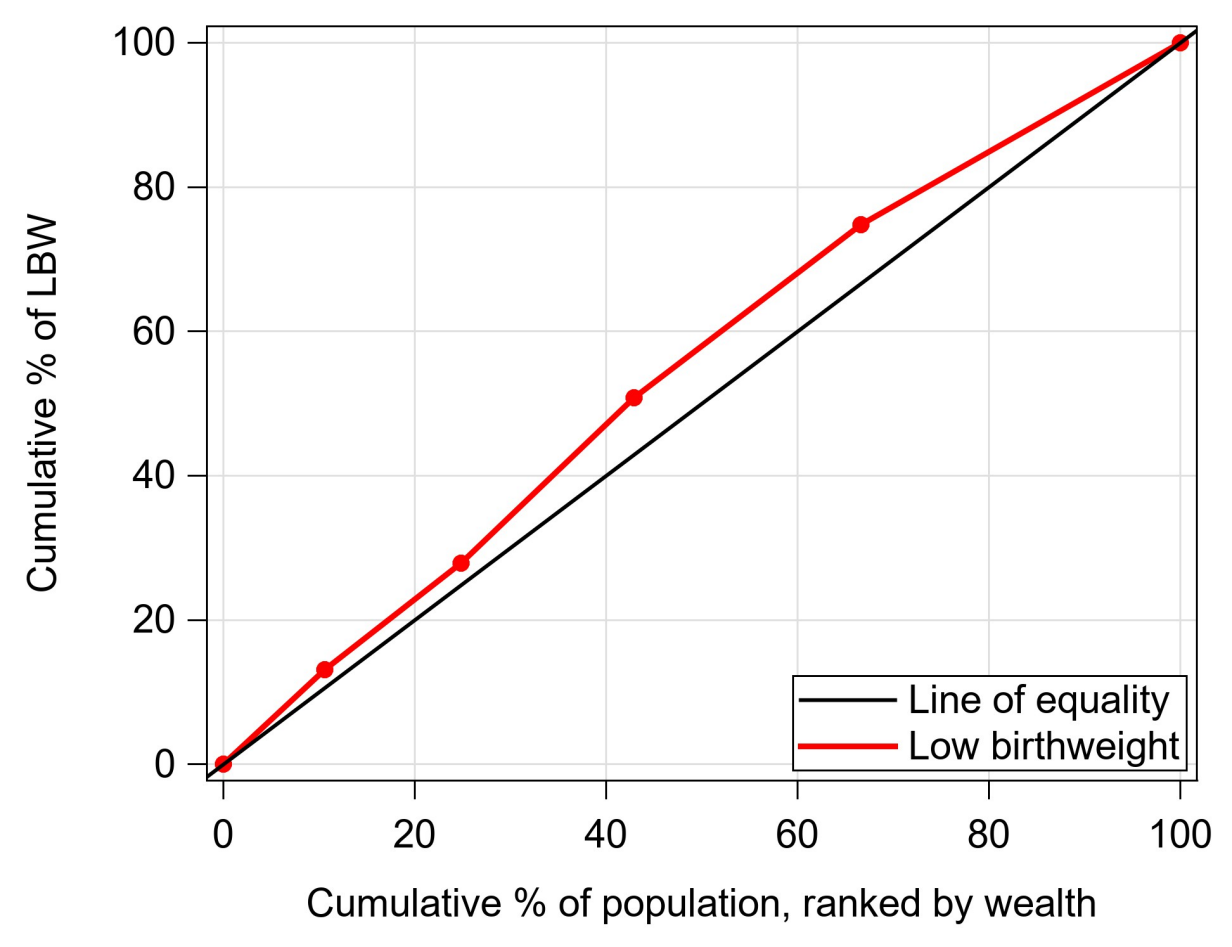
Concentration curve for LBW babies ranked by wealth in Bangladesh.

Fig 3 depicts the concentration curve for LBW rate ranked by wealth index. The CC showed that the line for LBW was above the line of equality, which indicated that the LBW babies were strongly concentrated in low socioeconomic groups (poorest). To clarify the result, the value of C-index was also presented in Table 2. The value of C-index: -0.096 (SE: 0.029; *P* = 0.029) demonstrated that there was a higher concentration of babies with LBW among mothers living in the lowest wealth quintile.

**Table 2:** Value of the concentration index.

Concentration index	Standard Error	95% CI	P-value
**-0.096**	0.029	(-0.175, -0.016)	0.029

Table 3 represents the prevalence and concentration index of LBW used by wealth quintile across the divisions in Bangladesh. The Sylhet division showed the highest C-index of -0.066, followed by the other divisions. LBW was more concentrated amongst the poorest socioeconomic households in the Sylhet division.

**Table 3:** Prevalence and concentration index of LBW used by wealth index across the divisions.

Wealth index	Division							
	Barisal	Chittagong	Dhaka	Khulna	Mymensingh	Rajshahi	Rangpur	Sylhet
Poorest	13.04	35.29	30.77	4.55	14.29	28.57	17.50	33.33
Poorer	14.29	8.33	16.67	16.67	8.77	19.05	10.17	12.50
Middle	21.15	30.77	16.33	25.71	10.42	6.82	8.51	20.83
Richer	12.50	21.05	17.71	9.72	7.14	14.47	8.89	20.51
Richest	2.70	13.99	9.28	6.82	8.51	14.00	8.20	17.20
Concentration Index	-0.166	-0.136	-0.174	-0.1605	-0.093	-0.109	-0.168	-0.066

#### Unadjusted and adjusted logistic regression analysis

Table 4 represents the results of unadjusted and adjusted LR analyses to identify the potential risk factors for LBW. The unadjusted LR showed that mothers from the poorest (COR: 2.032, 95% CI: 1.362–1.3.031, *P*<0.001) and middle-class (COR: 1.830, 95% CI: 1.289–2.598, *P*<0.001) households were more likely to have LBW babies compared to the mothers who were from the richest households. After adjusting for other confounders in the MLR model, mothers from the poorest families (AOR: 1.653, 95% CI: 0.969–2.820, *P* = 0.044) and middle income households (AOR: 1.561, 95% CI: 1.043–2.334, P = 0.030) were more likely to deliver LBW babies than the richest mothers. In unadjusted LR, mothers aged >40 years exhibited almost 5-fold higher odds of having LBW babies compared to the 21–30 years old mothers (COR: 4.664, 95% CI: 1.462–14.884, *P* = 0.009); whereas in adjusted LR, they showed almost 4-fold higher odds of having LBW babies (AOR: 3.963, 95% CI: 1.098–14.305, *P* = 0.036). Simple LR analysis demonstrated 2.227 times, 2.28 times and 74% higher likelihood of having LBW babies among the mothers who were from Sylhet (COR: 2.280, 95% CI: 1.299–4.003, *P* = 0.004), Chittagong (COR: 2.277, 95% CI: 1.363–3.806, *P* = 0.002), and Rajshahi (COR: 1.743, 95% CI: 1.002–3.035, *P* = 0.049) division, respectively, compared to those who were from Mymensingh division. Likewise, adjusted LR revealed that mothers who lived in the Sylhet (AOR: 2.900, 95% CI: 1.598–5.264, P<0.001), Chittagong (AOR: 2.813, 95% CI: 1.632–4.848, *P*<0.001), Rajshahi (AOR: 2.048, 95% CI: 1.156–3.628, *P* = 0.014), and Dhaka (AOR: 1.842, 95% CI: 1.486–3.356, *P* = 0.019) division were more likely to have babies with LBW compared to mothers who lived in Mymensingh division. Both unadjusted and adjusted LR analyses showed that the mothers who had no education, primary education, and completed secondary education were more likely to give birth to LBW babies than the higher educated mothers. Maternal height was found to be a significant risk factor for LBW.

**Table 4:** Unadjusted and adjusted LR analysis of risk factors for LBW.

Risk factors	Unadjusted LR				Adjusted LR			
	COR	95% CI of COR		P-value	AOR	95% CI of AOR		P-value
		Lower	Upper			Lower	Upper	
**Wealth index**								
Richest^®^	1.000				1.000			
Poorest	2.032	1.362	3.031	0.0005	1.653	0.969	2.820	0.044
Poorer	1.244	0.824	1.877	0.2987	1.044	0.632	1.724	0.8679
Middle	1.830	1.289	2.598	0.0007	1.561	1.043	2.334	0.0303
Richer	1.386	0.984	1.953	0.0618	1.172	0.805	1.705	0.4073
**Maternal age (Years)**								
21–30 ^®^	1.00				1.00			
< = 20	1.208	0.931	1.567	0.1558	1.119	0.842	1.486	0.4390
31–40	1.002	0.662	1.518	0.9921	1.033	0.647	1.647	0.8929
>40	4.664	1.462	14.884	0.0093	3.963	1.098	14.305	0.0355
**Division**								
Mymensingh^®^	1.00				1.00			
Barisal	1.467	0.804	2.677	0.2117	1.512	0.810	2.823	0.1939
Chittagong	2.277	1.363	3.806	0.0017	2.813	1.632	4.848	0.0002
Dhaka	1.501	0.891	2.528	0.1267	1.842	1.486	3.356	0.0193
Khulna	1.472	0.855	2.534	0.1635	1.751	0.995	3.079	0.0519
Rajshahi	1.743	1.002	3.035	0.0494	2.048	1.156	3.628	0.0140
Rangpur	1.219	0.695	2.138	0.4902	1.217	0.679	2.180	0.5094
Sylhet	2.280	1.299	4.003	0.0041	2.900	1.598	5.264	0.0005
**Maternal education**								
Higher^®^	1.00				1.00			
No education	1.212	0.3327	9.2399	0.0024	1.358	0.620	2.973	0.043
Primary	1.143	0.1929	8.8619	0.0029	1.27	0.582	1.526	0.8080
Secondary	1.042	0.1522	6.9159	0.0085	1.08	0.689	1.416	0.9483
**Husband’s education**								
Higher^®^	1.00				1.00			
No education	1.121	0.2518	4.387	0.0362	1.281	0.422	1.445	0.4315
Primary	1.183	0.1724	12.534	0.0004	1.186	0.710	1.662	0.7026
Secondary	1.020	0.1599	11.616	0.0007	1.074	0.813	1.695	0.3913
**Height**	0.994	0.991	0.996	< .0001	0.994	0.992	0.997	< .0001
**Weight**	0.998	0.997	0.999	0.0002	1.000	0.998	1.001	0.6931
**Parity**								
≤3	1.00				1.00			
>3	1.719	1.103	2.682	0.0168	1.175	0.675	2.044	0.5692
**Age at 1**^**st**^ **birth (years)**								
>25^®^	1.00				1.00			
<15	3.800	1.658	8.706	0.0016	2.773	1.112	6.916	0.0287
15–25	2.250	1.169	4.333	0.0152	1.728	0.856	3.492	0.1272
**Antenatal care initiation at 1**^**st**^ **trimester**								
Yes^®^	1.00				1.00			
No	1.387	1.087	1.770	0.0084	1.160	0.881	1.526	0.2896
**Number of antenatal visits**								
<4^®^	1.00				1.00			
≥4	1.323	1.028	1.702	0.0298	1.022	0.771	1.355	0.8783
**Delivery by CS**								
Yes^®^	1.00				1.00			
No	1.319	1.032	1.687	0.0272	1.098	0.839	1.439	0.4957
**Child is alive**								
Yes^®^	1.00				1.00			
No	2.993	1.563	5.732	0.0009	2.323	1.174	4.596	0.0155
**Toilet facility**								
Hygienic^®^	1.00				1.00			
Unhygienic	1.368	1.013	1.847	0.0410	1.091	0.763	1.560	0.6328
**Newspaper**								
Yes^®^	1.00				1.00			
No	1.576	1.104	2.249	0.0123	1.130	0.753	1.696	0.5564

COR: Crude odds ratio; OR: odds ratio; ®: Reference category.

Unadjusted LR showed that mothers who gave their first birth at age <15 years (COR: 3.800, 95% CI: 1.658–8.706, *P* = 0.002) or between age 15–25 years (COR: 2.250, 95% CI: 1.169–4.333, *P* = 0.015) had respectively 4-fold and 2-fold higher odds of giving birth to LBW babies than those who had their 1^st^ babies at the age >25 years. Similarly, adjusted LR revealed that women who were <15 years old at 1^st^ birth (AOR: 2.773, 95% CI: 1.112–6.916, *P* = 0.029) were more prevalent to give birth to babies with LBW than >25 years old mothers at 1^st^ birth. Mothers who lost their previous child were found to have higher odds of LBW babies compared those whose previous child was alive. Similarly, parity, antenatal care initiation at 1^st^ trimester, number of antenatal visits, delivery by CS, the child is alive, toilet facility, and newspaper reading habit were also statistically significant risk factors of LBW.

**Fig 4:**
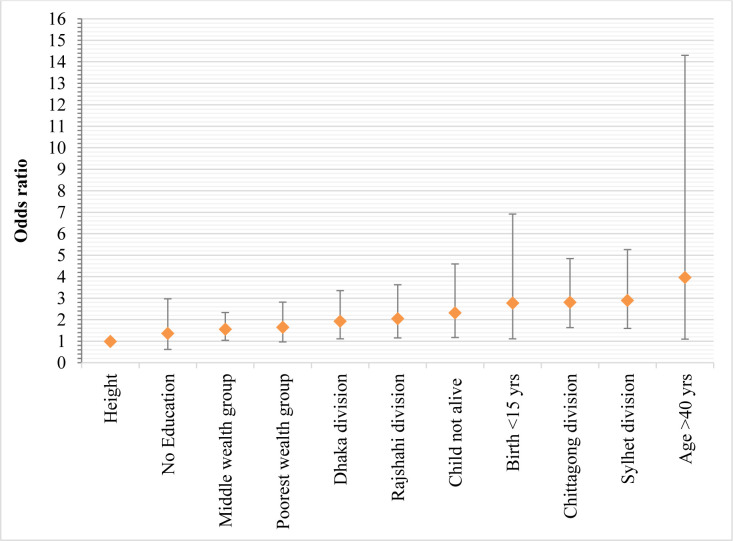
The odds ratio for significantly associated determinants of LBW.

Fig 4 represents the results of the adjusted odds ratios of the significant groups of significant risk factors of LBW. Mothers with age >40 years exhibited the strongest association with LBW (AOR: 3.963, 95% CI: 1.098–14.305, *P* = 0.0355) followed by the mothers who lived in Sylhet (AOR: 2.900, 95% CI: 1.598–5.264, *P* = 0.0005) and Chittagong (AOR: 2.813, 95% CI: 1.632–4.848, *P* = 0.0002) division.

## Discussion

The present study aimed to investigate the socioeconomic inequality in the prevalence of LBW among singleton births in Bangladesh and to identify the significant determinants using the latest BDHS (2017–18) data. We found that the overall prevalence of LBW among singleton births in our study population was 14.27%, which is much higher than other countries, including Russia (6.0%), Germany (7.0%), and Japan (10.0%) [47]. Lorenz curve and the Gini coefficient are widely used statistical tools for measuring health inequality when the study population is ordered by the health variable being studied. However, their main disadvantage is that they overlook the socioeconomic dimension [48, 49]. When the study population is ordered by socioeconomic status, the CC and C-index are useful statistical techniques, which incorporate the social dimension. Thus, our study used the C-index and CC to quantify the degree of socioeconomic inequality in LBW in Bangladesh. Our analysis showed that the occurrence of LBW was inequitably distributed among the socioeconomic groups, with a higher concentration of LBW infants among mothers living in the poorest households. Similar findings were reported by Mallick [50] and Khan et al. [28]. To the best of our knowledge, no study had investigated LBW in relation to socioeconomic inequality in Bangladesh. However, similar studies in the United States, United Kingdom, Canada, and Australia revealed increased risk of LBW delivery among women from poor families [51]. Also, similar findings of socioeconomic inequality were documented for other outcomes, including child malnutrition, and mother’s underweight, overweight, and obesity in Bangladesh [43, 52–54] and other low and middle-income countries [55–57]. Additionally, the results of the C-index stratified by divisions revealed that the concentration of LBW infants varies from region to region. Among all divisions in Bangladesh, the Sylhet division exhibited the highest C-index, where the occurrence of LBW was highly concentrated amongst the mothers who belonged to the poorest households.

Unadjusted and adjusted binary logistic regression analysis found that the wealth index is a critical gradient because women from the poorest households were at a higher odds of having babies with LBW than those from the richest households. Earlier studies showed similar findings for LBW and other outcomes (noncommunicable diseases and underweight) in LMICs [58–61]. Women living in the lowest socioeconomic group, who are the most food insecure are more likely to be malnourished [62] and are less likely to receive proper care during pregnancy, conditions that increases the risk of having a LBW infant [63].

Specific to Bangladesh, our study indicates that women who live in eastern divisions (Sylhet and Chittagong divisions) are more likely to give birth to LBW infants. Similar findings were reported by Khan et al. [29]. In the hilly areas, the government should take the necessary steps to reduce the number of births of LBW babies. We also found that maternal education level is an important determinant of LBW. In our study, women with no education had a higher odds of LBW infants compared to educated women. This is consistent with previous studies that found uneducated mothers are more likely to have LBW infants [64, 65]. Educated women are more likely to have high income and therefore able to make healthier choices including attending ANC, better nutrition, etc. Moreover, educated women are more aware of the available healthcare facilities and have a better knowledge of nutritional practices compared to uneducated women [66, 67]. Mother’s age was also a significant determinant of LBW in our study. The likelihood of LBW was higher among young mothers, which is consistent with the findings of other existing studies [68–70]. Mothers with an age at first birth <15 years were more likely to have LBW infants than mothers whose age at first birth is ≥15 years.

## Conclusion

The present study, based on BDHS 2017–18, revealed that singleton infants with LBW were more concentrated among mothers living in the poorest socioeconomic quintile in Bangladesh. Wealth index, maternal education level, maternal age, geographic region or administrative division, mother’s height, and maternal age at first birth were all significantly associated determinants of LBW. The highest risk of LBW was found among the infants born to uneducated women in the lowest socioeconomic quintile who lived in the eastern divisions (e.g., Sylhet and Chittagong) of Bangladesh. Intensive initiatives and efforts, by government and non-government organizations and agencies, to develop and implement policies and programs that addresses factors such as equitable access to health care, nutrition and education, focusing on communities at highest risk, are needed to reduce the prevalence of LBW infants in Bangladesh.

## Supporting information

S1 Table Distribution of the risk factors by wealth index using BDHS, 2017–18. (DOCX)
