# Supplementary material for: Socioeconomic inequality in the prevalence of low birth weight and its associated determinants in Bangladesh
Source: PLoS One. 2022 Oct 27;17(10):e0276718. doi: 10.1371/journal.pone.0276718 (PMC9612499; doi:10.1371/journal.pone.0276718)
Supplement: S1 Table — (DOCX) [file pone.0276718.s001.docx]

**S1 Table.** Distribution of the risk factors by Wealth index using BDHS, 2017-18.

|  | **Overall**  **n (%)** | **Poorest**  **n (%)** | **Poorer**  **n (%)** | **Middle**  **n (%)** | **Richer**  **n (%)** | **Richest**  **n (%)** | **P-value** |
| --- | --- | --- | --- | --- | --- | --- | --- |
| **Total** | 2138 (100) | 233 (10.90) | 298 (13.94) | 386 (18.05) | 508 (23.76) | 713 (33.35) |  |
| **Maternal age (Years)** |  |  |  |  |  |  |  |
| <=20 | 756 (35.36) | 107 (14.15) | 136 (17.99) | 155 (20.50) | 189 (25.00) | 169 (22.35) | <0.0001 |
| 21-30 | 1137 (53.18) | 110 (9.67) | 135 (11.87) | 194 (17.06) | 265 (23.31) | 433 (38.08) |  |
| 31-40 | 233 (10.90) | 16 (6.87) | 25 (10.73) | 33 (14.16) | 50 (21.46) | 109 (46.78) |  |
| >40 | 12 (0.56) | 0 (0.00) | 2 (16.67) | 4 (33.33) | 4 (33.33) | 2 (16.67) |  |
| **Residence** |  |  |  |  |  |  |  |
| Urban | 931 (43.55) | 32 (3.44) | 53 (5.69) | 104 (11.17) | 230 (24.70) | 512 (54.99) | <0.0001 |
| Rural | 1207 (56.45) | 201 (16.65) | 245 (20.30) | 282 (23.36) | 278 (23.03) | 201 (16.65) |  |
| **Division** |  |  |  |  |  |  |  |
| Barisal | 188 (8.79) | 23 (12.23) | 28 (14.89) | 52 (27.66) | 48 (25.53) | 37 (19.68) | <0.0001 |
| Chittagong | 312 (14.59) | 17 (5.45) | 24 (7.69) | 52 (16.67) | 76 (24.36) | 143 (45.83) |  |
| Dhaka | 376 (17.59) | 13 (3.46) | 24 (6.38) | 49 (13.03) | 96 (25.53) | 194 (51.60) |  |
| Khulna | 300 (14.03) | 22 (7.33) | 48 (16.00) | 70 (23.33) | 72 (24.00) | 88 (29.33) |  |
| Mymensingh | 243 (11.37) | 35 (14.40) | 57 (23.46) | 48 (19.75) | 56 (23.05) | 47 (19.34) |  |
| Rajshahi | 240 (11.23) | 28 (11.67) | 42 (17.50) | 44 (18.33) | 76 (31.67)) | 50 (20.83) |  |
| Rangpur | 292 (13.66) | 80 (27.40) | 59 (20.21) | 47 (16.10) | 45 (15.41) | 61 (20.89) |  |
| Sylhet | 187 (8.75) | 15 (8.02) | 16 (8.56) | 24 (12.83) | 39 (20.86) | 93 (49.73) |  |
| **Religion** |  |  |  |  |  |  |  |
| Muslim | 1917 (89.66) | 199 (10.38) | 267 (13.93) | 348 (18.15) | 456 (23.79) | 647 (33.75) | 0.235 |
| Non-Muslim | 221 (10.34) | 34 (15.38) | 31 (14.03) | 38 (17.19) | 52 (23.53) | 66 (29.86) |  |
| **Sex of child** |  |  |  |  |  |  |  |
| Male | 1159 (54.21) | 132 (11.39) | 159 (13.72) | 226 (19.50) | 257 (22.17) | 385 (33.22) | 0.179 |
| Female | 979 (45.79) | 101 (10.32) | 139 (14.20) | 160 (16.34) | 251 (25.64) | 328 (33.50) |  |
| **Maternal education** |  |  |  |  |  |  |  |
| No education | 57 (2.67) | 17 (29.82) | 12 (21.05) | 13 (22.81) | 9 (15.79) | 6 (10.53) | <0.0001 |
| Primary | 328 (15.34) | 69 (21.04) | 71 (21.65) | 59 (17.99) | 76 (23.17) | 53 (16.16) |  |
| Secondary | 1092 (51.08) | 124 (11.36) | 180 (16.48) | 228 (20.88) | 274 (25.09) | 286 (26.19) |  |
| Higher | 661 (30.92) | 23 (3.48) | 35 (5.30) | 86 (13.01) | 149 (22.54) | 368 (55.67) |  |
| **Husband education** |  |  |  |  |  |  |  |
| No education | 158 (7.39) | 46 (29.11) | 45 (28.48) | 27 (17.09) | 24 (15.19) | 16 (10.13) | <0.0001 |
| Primary | 513 (23.99) | 104 (20.27) | 121 (23.59) | 105 (20.47) | 116 (22.61) | 67 (13.06) |  |
| Secondary | 766 (35.83) | 61 (7.96) | 92 (12.01) | 163 (21.28) | 218 (28.46) | 232 (30.29) |  |
| Higher | 701 (32.79) | 22 (3.14) | 40 (5.71) | 91 (12.98) | 150 (21.40) | 398 (56.78) |  |
| **Height, cm [mean (SD)]** | 151.47 (5.73) | 150.03 (6.31) | 150.14 (5.79) | 151.05 (5.82) | 151.40 (5.28) | 152.77 (5.49) | <0.0001 |
| **Weight, kg [mean (SD)]** | 53.61 (10.90) | 48.23 (8.84) | 48.99 (8.69) | 51.56 (9.32) | 53.14 (9.95) | 58.75 (11.62) | <0.0001 |
| **BMI** | 23.32 (4.32) | 21.41 (3.69) | 21.72 (3.55) | 22.58 (3.78) | 23.15 (3.96) | 25.15 (4.64) | <0.0001 |
| Underweight | 265 (12.39) | 47 (17.74) | 59 (22.26) | 52 (19.62) | 61 (23.02) | 46 (17.36) | <0.0001 |
| Normal | 1192 (55.75) | 157 (13.17) | 190 (15.94) | 234 (19.63) | 292 (24.50) | 319 (26.76) |  |
| Overweight | 527 (24.65) | 25 (4.47) | 43 (8.16) | 85 (16.13) | 123 (23.34) | 251 (47.63) |  |
| Obese | 154 (7.20) | 4 (2.60) | 6 (3.90) | 15 (9.74) | 32 (20.78) | 97 (62.99) |  |
| **Parity** |  |  |  |  |  |  |  |
| ≤3 | 2013 (94.15) | 210 (10.43) | 280 (13.91) | 357 (17.73) | 474 (23.55) | 692 (34.38) | 0.0004 |
| >3 | 125 (5.85) | 23 (18.40) | 18 (14.40) | 29 (23.20) | 34 (27.20) | 21 (16.80) |  |
| **Age at 1^st^ birth (years)** |  |  |  |  |  |  |  |
| <15 | 81 (3.79) | 22 (27.16) | 19 (23.46) | 9 (11.11) | 22 (27.16) | 9 (11.11) | <0.0001 |
| 15-25 | 1914 (89.52) | 208 (10.87) | 271 (14.16) | 360 (18.81) | 456 (23.82) | 619 (32.34) |  |
| >25 | 143 (6.69) | 3 (2.10) | 8 (5.59) | 17 (11.89) | 30 (20.98) | 85 (59.44) |  |
| **Marriage to 1^st^ birth interval** |  |  |  |  |  |  |  |
| <=30 | 1513 (70.77) | 173 (11.43) | 232 (15.33) | 267 (17.65) | 344 (22.74) | 497 (32.85) | 0.0193 |
| >30 | 625 (29.23) | 60 (9.60) | 66 (10.56) | 119 (19.04) | 164 (26.24) | 216 (34.56) |  |
| **ANC initiation at 1^st^ trimester** |  |  |  |  |  |  |  |
| Yes | 1110 (51.92) | 84 (7.57) | 109 (9.82) | 180 (16.22) | 245 (22.07) | 492 (44.32) | <0.0001 |
| No | 1028 (48.08) | 149 (14.49) | 189 (18.39) | 206 (20.04) | 263 (25.58) | 221 (21.50) |  |
| **Number of antenatal visits** |  |  |  |  |  |  |  |
| <4 | 684 (31.99) | 109 (15.94) | 133 (19.44) | 130 (19.01) | 163 (23.83) | 149 (21.78) | <0.0001 |
| >=4 | 1454 (68.01) | 124 (8.53) | 165 (11.35) | 256 (17.61) | 345 (23.73) | 564 (38.79) |  |
| **During pregnancy iron tablet** |  |  |  |  |  |  |  |
| Yes | 1850 (86.53) | 183 (9.89) | 250 (13.51) | 332 (17.95) | 442 (23.89) | 643 (34.76) | 0.0001 |
| No | 288 (13.47) | 50 (17.36) | 48 (16.67) | 54 (18.75) | 66 (22.92) | 70 (24.31) |  |
| **Place of delivery** |  |  |  |  |  |  |  |
| Home | 198 (9.26) | 49 (24.75) | 50 (25.25) | 38 (19.19) | 40 (20.20) | 21 (10.61) | <0.0001 |
| Public sector | 528 (24.70) | 69 (13.07) | 81 (15.34) | 101 (19.13) | 125 (23.67) | 152 (28.79) |  |
| Private sector | 1235 (57.76) | 103 (8.34) | 147 (11.90) | 218 (17.65) | 288 (23.32) | 479 (38.79) |  |
| NGO sector | 175 (8.19) | 12 (6.86) | 19 (10.86) | 28 (16.00) | 55 (31.43) | 61 (34.86) |  |
| Other | 2 (0.09) | 0 (0.00) | 1 (50.00) | 1 (50.00) | 0 (0.00) | 0 (0.00) |  |
| **Delivery by CS** |  |  |  |  |  |  |  |
| Yes | 1341 (62.72) | 98 (7.31) | 163 (12.16) | 235 (17.52) | 302 (22.52) | 543 (40.49) | <0.0001 |
| No | 797 (37.28) | 135 (16.94) | 135 (16.94) | 151 (18.95) | 206 (25.85) | 170 (21.33) |  |
| **Child is alive** |  |  |  |  |  |  |  |
| Yes | 2095 (97.99) | 223 (10.64) | 291 (13.89) | 377 (18.00) | 501 (23.91) | 703 (33.56) | 0.064 |
| No | 43 (2.01) | 10 (23.26) | 7 (16.28) | 9 (20.93) | 7 (16.28) | 10 (23.26) |  |
| **Toilet facility ^a^** |  |  |  |  |  |  |  |
| Hygienic | 1770 (82.79) | 114 (6.44) | 179 (10.11) | 299 (16.89) | 470 (26.55) | 708 (40.00) | <0.0001 |
| Unhygienic | 368 (17.21) | 119 (32.34) | 119 (32.34) | 87 (23.64) | 38 (10.33) | 5 (1.36) |  |
| **Newspaper** |  |  |  |  |  |  |  |
| Yes | 383 (17.91) | 7 (1.83) | 20 (5.22) | 42 (10.97) | 76 (19.84) | 238 (62.14) | <0.0001 |
| No | 1755 (82.09) | 226 (12.88) | 278 (15.84) | 344 (19.60) | 432 (24.62) | 475 (27.07) |  |
| **Television** |  |  |  |  |  |  |  |
| Yes | 1621 (75.82) | 91 (5.61) | 173 (10.67) | 278 (17.15) | 425 (26.22) | 654 (40.35) | <0.0001 |
| No | 517 (24.18) | 142 (27.47) | 125 (24.18) | 108 (20.89) | 83 (16.05) | 59 (11.41) |  |

ANC: antenatal care, BMI: body mass index, CS: caesarean section, LBW: low birth weight, NGO: non-governmental organization, SD: standard deviation.

^a^ Hygienic toilet facility includes flush toilet, flush to piped sewer system, flush to septic tank, flush to pit latrine, flush to somewhere else, flush to unknow place, pit toilet latrine, ventilated improved pit latrine (VIP), pit latrine with slab and composting toilet. Unhygienic toilet facility includes all other toilet facilities that are not included under hygienic toilet facility (pit latrine without slab/open pit, no facility, no facility/bush/field, bucket toilet, hanging toilet/latrine and other).
